# Supplementary material for: ProteinShader: illustrative rendering of macromolecules
Source: BMC Struct Biol. 2009 Mar 30;9:19. doi: 10.1186/1472-6807-9-19 (PMC2672931; doi:10.1186/1472-6807-9-19)
Supplement: Additional file 1 — ProteinShader program without source code. This compressed file contains the complete ProteinShader program including associated libraries, but no source code. A README.txt file gives an overview of the ProteinShader distribution, and the index.html file in the help subdirectory has directions on getting started with the program as well as a set of tutorials. [file 1472-6807-9-19-S1.zip › ProteinShader-beta-0_9_4-binary/help/api/org/proteinshader/gui/listeners/WindowListenerFactory.html]

WindowListenerFactory (ProteinShader API)


|  |  |  |
| --- | --- | --- |
| **Overview** \| **Package** \| **Class** \| **Use** \| **Tree** \| **Deprecated** \| **Index** \| **Help** | |  |
| **PREV CLASS**   NEXT CLASS | **FRAMES**    **NO FRAMES**     **All Classes** |  |
| SUMMARY: NESTED \| FIELD \| CONSTR \| METHOD | DETAIL: FIELD \| CONSTR \| METHOD |  |


---


## org.proteinshader.gui.listeners Class WindowListenerFactory

```
java.lang.Object
  org.proteinshader.gui.listeners.WindowListenerFactory
```

---

``` public class WindowListenerFactory extends Object ```

Creates a window listener for closing the retractable control panel
that opens on the right-side of the GUI.

---

**Constructor Summary**

`WindowListenerFactory(Mediator mediator)`
  
          Constructs a WindowListenerFactory.


| **Method Summary** | |
| --- | --- |
| `WindowListener` | `createControlFrameListener()`             Creates a window listener for the spring-loaded frame that holds the control panel. |

**Methods inherited from class java.lang.Object**

`clone, equals, finalize, getClass, hashCode, notify, notifyAll, toString, wait, wait, wait`

**Constructor Detail**

### WindowListenerFactory

```
public WindowListenerFactory(Mediator mediator)
```

:   Constructs a WindowListenerFactory.

    **Parameters:**: `mediator` - the centralized Mediator object that listeners call on to accomplish their task.


**Method Detail**

### createControlFrameListener

```
public WindowListener createControlFrameListener()
```

:   Creates a window listener for the spring-loaded frame that holds
    the control panel. When the frame's close box is clicked, the
    spring-loaded frame will be closed using animation.

    :   **Returns:**: The window listener.


---


|  |  |  |
| --- | --- | --- |
| **Overview** \| **Package** \| **Class** \| **Use** \| **Tree** \| **Deprecated** \| **Index** \| **Help** | |  |
| **PREV CLASS**   NEXT CLASS | **FRAMES**    **NO FRAMES**     **All Classes** |  |
| SUMMARY: NESTED \| FIELD \| CONSTR \| METHOD | DETAIL: FIELD \| CONSTR \| METHOD |  |


---

# *Copyright © 2007-2008*
